# Unwelcome guests – the role of gland-associated *Helicobacter pylori* infection in gastric carcinogenesis

**DOI:** 10.3389/fonc.2023.1171003

**Published:** 2023-04-21

**Authors:** Giulia Beccaceci, Michael Sigal

**Affiliations:** ^1^ Medical Department, Division of Gastroenterology and Hepatology, Charité-Universtitätsmedizin Berlin, Berlin, Germany; ^2^ The Berlin Institute for Medical Systems Biology (BIMSB), Max Delbrück Center for Molecular Medicine, Berlin, Germany

**Keywords:** infection, gastric cancer, epithelial stem cells, gland-associated bacteria, gland-colonization

## Abstract

*Helicobacter pylori* (*H. pylori*) are Gram-negative bacteria that cause chronic gastritis and are considered the main risk factor for the development of gastric cancer. *H. pylori* have evolved to survive the harsh luminal environment of the stomach and are known to cause damage and signaling aberrations in gastric epithelial cells, which can result in premalignant and malignant pathology. As well as colonizing the gastric mucus and surface epithelial cells, a subpopulation of *H. pylori* can invade deep into the gastric glands and directly interact with progenitor and stem cells. Gland colonization therefore bears the potential to cause direct injury to long-lived cells. Moreover, this bacterial subpopulation triggers a series of host responses that cause an enhanced proliferation of stem cells. Here, we review recent insights into how gastric gland colonization by *H. pylori* is established, the resulting pro-carcinogenic epithelial signaling alterations, as well as new insights into stem cell responses to infection. Together these point towards a critical role of gland-associated *H. pylori* in the development of gastric cancer.

## 1 Introduction

*H. pylori* were first identified in 1984 by Barry Marshall and Robin Warren as bacteria present in the stomach of patients with chronic gastritis (1). The correlation between *H. pylori* infection and gastric tumorigenesis has been extensively investigated in the last decades (2, 3). In fact, almost 90% of gastric cancers can be traced back to the pathogenic effects of *H. pylori* (4), and eradication of the infection strongly correlates with a reduced risk of gastric cancer development. For these reasons, *H. pylori* infection is considered the main risk factor for gastric cancer and has been classified as a class I carcinogen by the World Health Organization (WHO) since 1994 (5).

*H. pylori* are spiral-shaped, microaerophilic Gram-negative bacteria that colonize the human stomach. Although ubiquitous across the globe, the prevalence of infection varies significantly, ranging on average from more than 70% of individuals living in developing countries to less than 40% in Western countries (6). In most cases, *H. pylori* infection causes asymptomatic chronic gastritis and persists lifelong. However, a small percentage of infected individuals will develop more severe pathologies, such as peptic ulceration (10-20%) (7), B cell mucosa-associated lymphoid tissue (MALT) lymphoma and gastric cancer (up to 2%). The occurrence of severe pathology has been correlated with strain virulence, host genetics, as well as environmental factors (8, 9). *H. pylori* strains can be classified into two main groups, depending on whether they carry the cytotoxin-associated gene pathogenicity island (cagPAI), a DNA region that encodes virulence factors, including the most studied cytotoxin-associated gene A (CagA) (10). CagA has been classified as an oncogenic protein, since individuals infected with CagA-negative strains are less likely to develop neoplastic lesions, although they will still suffer from gastritis (11).

Gastric cancer is the fifth most frequent cancer worldwide (12). The first formal description of the cascade of events that lead to gastric cancer onset was proposed by Pelayo Correa in 1975 (13). Briefly, inflammation can occur in the normal gastric mucosa, leading to gastritis. Gastritis can then progress to multifocal atrophic gastritis, a condition defined by markedly reduced glandular tissue. This is considered the first step of the precancerous cascade, which is followed by intestinal metaplasia, dysplasia and ultimately gastric cancer development (13). The immune system is known to play an important role in gastric mucosa pathology, including in the context of *H. pylori* infection, and immunological responses have been extensively investigated and described previously (14–17). Several classifications of gastric cancer have been developed, including one proposed by the Asian Cancer Research Group (ACRG), with the aim of identifying genomic alteration and mutational signatures in gastric cancer patients (18). Additionally, the Cancer Genome Atlas Research Network (TCGA) published a classification of gastric cancer into four main subtypes according to the tumor molecular profile: tumors positive for Epstein–Barr virus, microsatellite unstable tumors, genomically stable tumors and tumors with chromosomal instability (19).

The ability of *H. pylori* to colonize and directly interact with the epithelium is crucial to the pathogenesis caused by these bacteria. Imaging studies have demonstrated that *H. pylori* are found in the gastric mucus layer in close association with mucous pit cells and *H. pylori* have been found to possess specific molecular mechanisms to locate these cells (20). However, pit cells are short-lived and terminally differentiated, thus their interplay with *H. pylori* is unlikely to cause long-term pathology. Instead, it has been shown that a subset of bacteria can invade deep into the gastric glands, where they interact with longer-lived progenitor and stem cells (21, 22). On one hand, these gland-associated microcolonies of *H. pylori* are now believed to represent a reservoir of proliferative bacteria that enable the persistence and expansion of the infection (23). On the other hand, recent discoveries suggest that the interaction of *H. pylori* with the long-lived stem cells residing in the glands bears unique pathogenic potential. In particular, *H. pylori*, *via* direct and indirect mechanisms, dysregulate important signaling pathways involved in gastric stem cell niche maintenance, leading to severe hyperplasia and setting the basis for malignant progression.

Here, we discuss recent insights into gland-associated *H. pylori*, focusing on their relevance for the establishment of a persistent infection and the ability of the bacteria to interfere with cellular signaling in the epithelium, which suggests that gland-associated *H. pylori* are key drivers of gastric pathology.

## 2 *H. pylori* exploit specific features that allow gland colonization

*H. pylori* exploit different strategies to survive in the harsh environment of the stomach. One key feature is the production of urease, which enables localized neutralization of the highly acidic stomach lumen, while flagella-mediated motility and helical morphology allow bacteria to penetrate the mucous layer (24). *H. pylori* express several chemoreceptors, such as TlpA, TlpB, TlpC and TlpD. TlpB is able to sense the chemoattractant urea, and activation of the downstream signaling is thought to be important for sensing epithelial cells (20, 25). TlpA, TlpB and TlpD act as acid sensors, with acid being a chemorepellent. TlpD is located in the cytoplasm and inner membrane and can transduce the signal to the flagellar machinery *via* CheA kinase activation, thus leading to changes in bacterial trajectory in response to local lower pH (26–28). TlpC, on the other hand, is able to sense lactate, a cell-metabolic product that acts as a chemoattract (29). The activity of chemoreceptors is therefore fundamental to guide bacteria to the protective and nutrient-rich mucous layer that covers the epithelium. Moreover, the intact chemotactic apparatus is also important for gland colonization. Mutant *H. pylori* lacking TlpD or other chemotactic proteins, such as ChePep and CheY, were shown to have a significantly reduced ability to colonize gastric glands and were outcompeted by wild-type counterparts with intact chemotaxis machinery, thus suggesting that gland colonization is beneficial for long-term persistence (22, 25, 30, 31). Once it has penetrated the mucous layer, *H. pylori*’s survival and proliferation are determined by its ability to colonize gastric epithelial cells. Specifically, *H. pylori*’s outer membrane is enriched with proteins that mediate its specific adhesion to epithelial cells. The presence of these adhesins, such as BabA, SabA, OipA, HopQ and HomB, has also been associated with an increased risk of gastric cancer development in clinical studies (32–34).

Although a large number of *in vitro* studies have explored its aberrant effects on host cells, for a long time it was not clear which cells can be targeted by *H. pylori in vivo*. In a recent publication, Aguilar and colleagues showed that *H. pylori* are capable of binding with high affinity to differentiated gastric pit cells expressing high levels of gastrokine 1 and 2 (GKN1-GKN2) (20). This is in line with clinical studies showing that in human samples *H. pylori* is also detected in that region. However, pit cells are short-lived and are replaced every 2-3 days (35).

Recent studies have identified that a subset of *H. pylori* can penetrate deep into the base of gastric glands where long-lived cells, such as stem and progenitor cells, reside (21, 22). Furthermore, Keilberger and colleagues showed that in mice the highest bacterial number in gastric glands is observed between 2 to 4 weeks after inoculation, while in the chronic phase of the infection only a few glands were still colonized, and only with a small number of bacteria. Exploiting two fluorescently-labeled *H. pylori* strains, the authors additionally showed that a primary infection is protective against a secondary infection (30). In order to investigate the dynamics of *H. pylori* gland colonization, Fung and colleagues co-infected mice with two fluorescently-labeled strains at equal ratios. They observed that the number of glands colonized with only a single strain was significantly higher than the number of glands containing a mixture of both strains. Moreover, even two months after inoculation, the gland-associated bacterial populations did not become more heterogeneous, but instead retained their identity and only expanded in size. These findings suggest that gland-colonizing bacteria can create a protective niche that enables their survival and proliferation. Fung and colleagues propose that, once this close interaction is established, gland-associated bacteria may represent an important reservoir that can act as a source for replenishing the surface bacteria populations, thus expanding the area infected by a single bacteria strain until all permissive niches are colonized (23).

## 3 Direct effects of *H. pylori* on epithelial cells

Bacteria can secrete pathogenic factors that are responsible for signaling alterations and ultimately aberration of cell behavior. Among the most studied is vacuolating cytotoxin A (VacA), secreted *via* a type V auto transport secretion system (36). Upon binding to host cells and subsequent internalization, VacA causes extensive vacuolation and damage to mitochondria, altering mitochondrial membrane permeability and inducing cytochrome c release (37). Moreover, it has been shown that VacA can alter cell metabolism, specifically inducing a shift to catabolic metabolism *via* mTORC1 inhibition (38). In addition, it can activate intracellular signaling, such as the p38 pathway - however, the exact mechanisms and consequences of this are not yet fully understood (39).

*H. pylori* can also translocate virulence factors directly into host cells, such as CagA and metabolic precursors of bacterial lipopolysaccharide (LPS), namely ADP-β- d- manno-heptose (ADP-heptose) and d- glycerol-β- d- manno- heptose 1,7- bisphosphate (HBP) (40) *via* the type IV secretion system (TFSS). The TFSS consists of a “needle-and-syringe” mechanism that allows the direct transfer of bacterial factors into the host cytoplasm (41). ADP-heptose has been described as a potent activator of NF-κB signaling in infected cells *via* the ALPK1-TIFA signaling pathway (42). Both the TFSS and CagA have been epidemiologically linked to gastric cancer, since carrying strains are associated with more severe clinical outcomes (43, 44). Given the correlation between CagA protein and gastric carcinogenesis, its effects have been extensively investigated. Once injected into host cells, CagA is activated *via* phosphorylation by host phosphatases and initiates a series of signaling cascades. CagA is able to bind SHP2, an oncogenic phosphatase that aberrantly activates ERK signaling (45). Moreover, CagA activation has been linked to the ability of *H. pylori* to inhibit the activity of tumor suppressor genes, such as p14ARF (46) [which is an important factor for regulating the cell cycle *via* p53-dependent or independent signaling (47) as well as for oncogenic stress responses (48)] and the pro-apoptotic factor Siva1 (49). The anti-apoptotic effect of *H. pylori* infection has been extensively investigated and linked to both direct and indirect effects of CagA (50–52), such as CagA-dependent upregulation of the apoptosis inhibitor cIAP2 (53). In addition, CagA has been shown to cause dysregulation of the cytoskeleton and cell polarity after interacting with proteins involved in tight junctions and cell membrane integrity, such as ZO-1, E-cadherin and claudin-7 (54, 55). This effect has been shown to promote an invasive phenotype in gastric epithelial cells. Indeed, Bagnoli and colleagues observed that CagA can cause loss of apicobasal polarity of gastric epithelial cells, which acquire migratory features characteristics of epithelial-to-mesenchymal transition (56).

Interestingly, different studies have highlighted that *H. pylori* are able to interfere with biomolecule availability in host cells. For instance, Tan and colleagues reported that CagA can induce alterations in iron trafficking, altering cell polarity and favoring iron acquisition by the bacteria. Mongolian gerbils fed on an iron-replete diet and infected with *H. pylori* WT or ΔCagA (which have a defective injection system) did not show different colonization levels. However, in animals fed on an iron-reduced diet, the ΔCagA bacteria´s ability to colonize the stomach was significantly impaired (57). Another study showed that Mongolian gerbils fed on an iron-reduced diet were more susceptible to infection with *H. pylori* with regards to tumor formation. In these animals, gland colonization was increased, single bacteria showed increased expression of TF4SS and consequently more CagA translocation, thus resulting in accelerated tumor formation. This suggests that gland colonization and CagA translocation support bacterial iron uptake *in vivo*, while simultaneously initiating aberrant pro-carcinogenic effects in the epithelium (58). Moreover, *H. pylori* were shown to obtain cholesterol from infected cells *via* the expression of cholesterol-α-glucosyltransferase (59). The resulting cholesterol depletion in infected cell membranes inhibits IFN-γ signaling, enabling the bacteria to evade the host immune system. Indeed, areas with high gland colonization levels showed local immunosuppression and decreased expression of IFN-γ target genes, suggesting that gland colonization might also be an important mechanism to interfere with inflammatory, antimicrobial tissue responses.

Importantly, in addition to inducing potentially pro-oncogenic signaling, *H. pylori* infection can induce DNA damage in infected cells, *via* CagA-dependent and independent mechanisms. CagA-carrying strains favor the occurrence of double-strand breaks (DSBs) *via* siRNA-mediated downregulation of Rad51 and *via* inhibition of PAR1b, a kinase necessary to phosphorylate BRCA1. Unphosphorylated BRCA1 is then unable to translocate into the nucleus and exert its function as part of the DSB repair machinery (60, 61). These effects lead to replication fork instability and incorrect repair of DSBs, potentially causing irreversible DNA damage. Moreover, CagA-positive *H. pylori* strains increase Reactive Oxygen Species (ROS) production both in immune and epithelial cells, further enhancing the probability of mutagenic events (45). Notably, the occurrence of DSBs was shown to be tightly linked to the presence of a functional T4SS and NF-κB transcriptional activity. Hartung and colleagues revealed that DSBs can occur due to the activity of the nucleotide excision repair (NER) endonucleases XPG and XPF and that this is dependent on the presence of a functional cagPAI (62). In addition, they observed that DSBs amplify the transcription of NF-κB target genes. Interestingly, Koeppel and colleagues demonstrated that DNA damage upon *H. pylori* infection does not happen randomly throughout the genome, but that telomeric and transcribed genomic regions had a higher probability of acquiring genetic mutations. These patterns correlated with mutations that are frequently observed in gastric cancer patients, thus strengthening the idea that genetic alterations necessary for tumor development might be directly induced by *H. pylori* (63).

## 4 Gastric stem cells and gland responses to *H. pylori* infection

**Figure 1:**
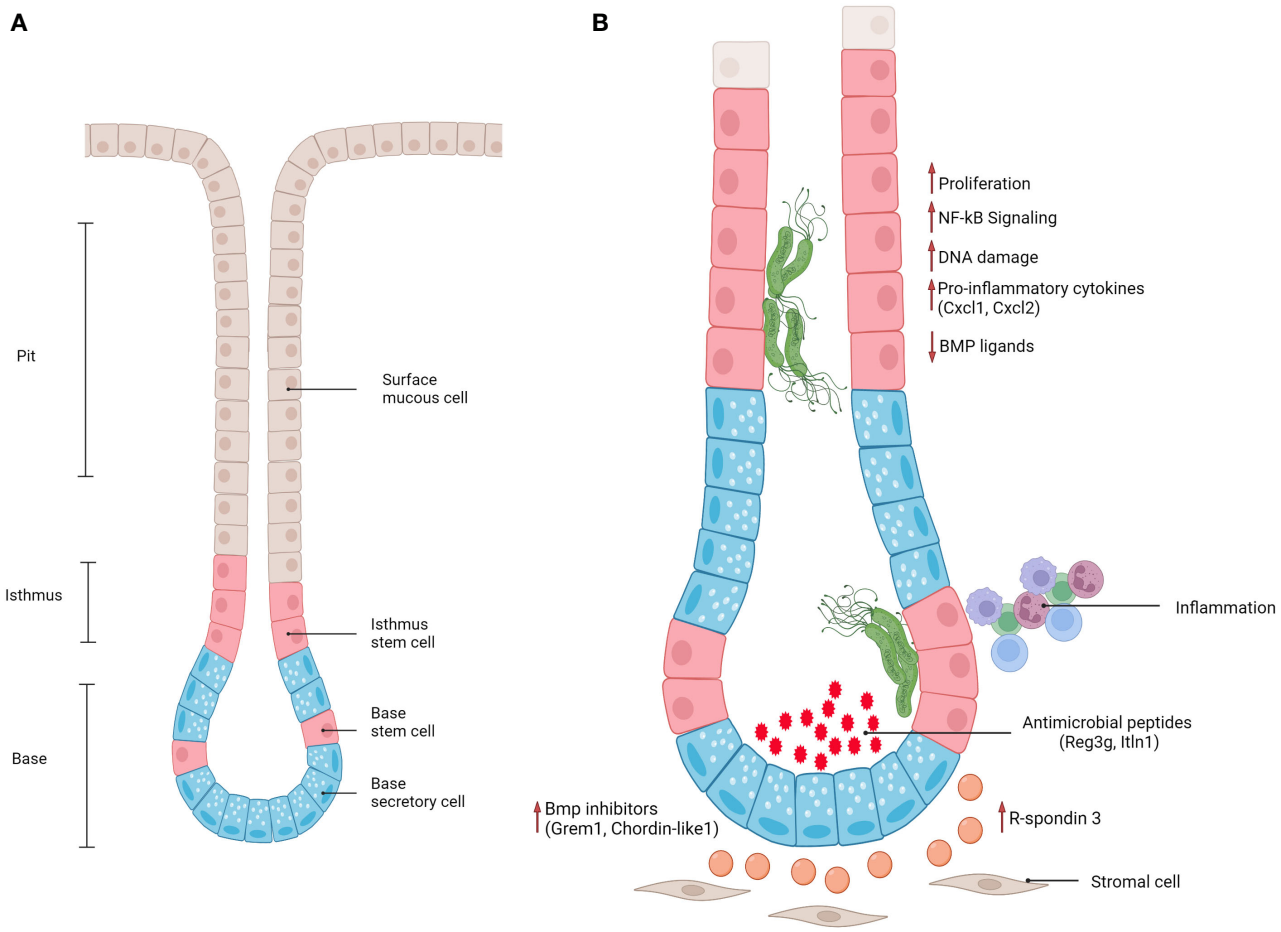
**(A)** Schematic representation of antral gland regions (pit, isthmus, base) and epithelial cell populations. **(B)** Schematic representation of epithelial and stromal responses in antral gastric glands infected with gland-associated *H. pylori*.

The stomach wall consists of 5 layers: serosa (outermost), subserosa, the muscle layer, submucosa and mucosa (innermost). The mucosa is in contact with the lumen of the stomach, and is formed by deep epithelial invaginations, called glands, as well as pits at the surface of the epithelium (64). The glands can be further divided into isthmus (where proliferative progenitor cells reside), neck and base (**Figure 1A**). Gastric glands display a compartmentalized structure along their lumen-to-base axis, with terminally differentiated pit cells at the surface being continually shed into the lumen and replacement cells produced by the proliferation and differentiation of stem and progenitor cells in the base and isthmus. Pit cells are replaced on average every 3 days (35), while parietal and chief cells usually have a life span of 2 and 6 months, respectively (65). Proper regulation of proliferation and differentiation of stem and progenitor cells is therefore crucial for maintaining a functional tissue.

Although the macroscopic structure of the stomach is highly conserved, it consists of different regions that harbor distinct cell populations (**Figure 1A**). For instance, only antral glands contain the gastrin-secreting enteroendocrine cells (G-cells) that promote acid secretion. Corpus glands contain chief cells and acid-secreting parietal cells, which are mostly absent in the antrum. In both regions, the gland pit contains terminally differentiated cells that produce mucus, which is important to protect the epithelium from the toxic, highly acidic environment of the lumen (66). Between antrum and corpus, a “transitional zone” can be identified, which presents mixed features of both (67).

Many studies have focused on the characterization of gastric stem cells. Again, antrum and corpus regions display substantial differences. Barker and colleagues showed that Lgr5+ cells located at the base of antral glands are multipotent and can renew the entire gland. They also showed that these Lgr5+ cells can generate gastric organoids *in vitro* (68). It has since been demonstrated that there is another population of highly proliferative Axin2+/Lgr5- stem cells, which is located in the lower isthmus region (69). These cells do not express any marker related to differentiated gastric cells and can repopulate the whole gland within 7 days. Several other markers of gland base and isthmus cells have been identified, which likely show an overlap with the two aforementioned populations [for a comprehensive overview see (70)]. The corpus similarly contains two regions that harbor distinct cells with a high regenerative capacity: the isthmus contains proliferative cells that are able to repopulate the glands (71). In addition, Stange et al. demonstrated the existence of a second stem cell reservoir marked by Troy+, at the base of corpus glands where chief cells are located (72). These cells rarely proliferate during homeostasis while upon damage they become proliferative and give rise to other differentiated cell populations (72). This concept has been challenged recently, suggesting that gland regeneration after injury is driven by mucous neck cells instead (73). Importantly, the idea that multiple lineages located in the isthmus and base compartments can act as stem cells according to different contexts (homeostasis or injury) has now gained a broader consensus, both in the antrum and in the corpus.

As described above, DNA damage is one effect caused by the direct interaction of *H. pylori* with host cells Interestingly, DSBs have been observed in gastric Lgr5+ cells in infected individuals (74). Moreover, Bauer and colleagues showed that *H. pylori*-driven DNA damage occurs preferentially in cells that are in S-phase and this also correlates with a functional NF-κB response upon pathogenic triggers, implicating that these events are more likely to occur in the progenitor/stem cell compartment of the gland (75).

Importantly, gastric glands are surrounded by stromal cells that secrete niche factors that are fundamental for orchestrating stem cell behavior. The stromal cells set up specific patterns and gradients of secreted growth factors along the lumen-to-base axis, which are important to maintain cell identity, proliferation and differentiation. Specifically, Wölffling and colleagues, exploiting polarized mucosoid cultures from primary human gastric cells, identified EGF, BMP and Noggin as key niche factors crucial for controlling cell fate in human gastric corpus glands. EGF and BMP are present at higher concentrations at the pit, while concentrations of Noggin are highest at the base (76).

Stromal myofibroblasts also secrete Wnt signaling molecules that drive stem cell turnover and play a fundamental role in regulating gastrointestinal homeostasis (77). Furthermore, multiple cell types can secrete Wnt ligands: innate lymphoid type 2 cells (ILC-2) were shown to be important producers of Wnt5a, which was able to promote tumor development *via* RhoA signaling activation (71). Indeed, in an *in vivo* model of *Helicobacter felis* infection, which is known to elicit a stronger inflammatory response than *H. pylori* in mice (78), Wnt5a was found to be fundamental for Mist+ stem cell activation and expansion upon infection (71).

Furthermore, BMP signaling was shown to be fundamental in the maintenance of cell identity along the lumen-to-base axis, with BMP inhibitors produced by stromal cells located at the base of the glands and BMP activators mostly present at the surface, where it is important for driving terminal differentiation of progenitor cells into Muc5ac+ mucous pit cells. Recently, Kapalczynska, Lin and colleagues showed that *H. pylori* can interfere with BMP signaling (79). In a murine model, the presence of *H. pylori* caused loss of Bmp ligand expression at the gland surface, as well as upregulation of BMP inhibitors in the base (**Figure 1**), leading to an accumulation of base cells and expansion of the proliferative compartment. This effect was dependent on CagA translocation into host cells, since mice infected with a TFSS-defective strain did not show these signaling alterations. However, which molecular pathways CagA interferes with to cause these alterations remains elusive. Similarly, another study showed that inhibition of BMP signaling *in vivo* causes a markedly enhanced inflammatory response, as well as accelerated development of dysplastic regions (80) suggesting that inhibition of BMP signaling upon infection can be viewed as a critical event in the context of gastric carcinogenesis.

One of the fundamental signaling pathways in gastrointestinal tract homeostasis is Wnt signaling. In the stomach specifically, R-spondin 3, a Wnt signaling enhancer, is produced by a subset of Mhy11+ myofibroblasts located at the base of the glands. R-spondin 3 has been shown to play an essential role in maintaining cell identity in the base and lower isthmus of gastric glands. Specifically, it is a key regulator of proliferation in the stem cell compartment (Axin2+/Lgr5-) in antral glands and of differentiation of secretory Lgr5+ cells in corpus glands during homeostasis (69, 81). Importantly, the stromal cell compartment not only orchestrates gland turnover during homeostasis but also controls epithelial regeneration after damage induced by injury or infection. Upon *H. pylori* infection of an *in vivo* mouse model, myofibroblasts located at the gland base express increased levels of R-spondin 3 in both antrum and corpus (69, 70, 81) (**Figure 1**). In the antrum, this causes an expansion of the proliferative compartment (characterized by proliferative Axin2+/Lgr5- progenitors), thus leading to gland hyperplasia. In addition, R-spondin 3 affects Lgr5+ base epithelial cells, inducing their differentiation into secretory cells that produce and secrete anti-microbial peptides, such as Reg3g and Itln1 (69, 70). This anti-microbial response counterbalances gland colonization, but fails to fully clear *H. pylori*.

In the corpus, on the other hand, exploiting R-spondin 3 knockout and knock-in mouse models, R-spondin 3 was shown to be important not only for boosting Wnt signaling but also for the activation of YAP signaling in the gland base upon infection with *H. pylori* (81). YAP is a transcription factor known to be involved in injury-driven epithelial regeneration of the gastrointestinal tract epithelium (82). Interestingly, *in vivo* murine models showed that chemical injury of epithelial gastric glands also causes a peak of R-spondin 3 production and YAP activation, both of which are downregulated within a matter of days once epithelial integrity is restored (81). However, upon *H. pylori* infection, R-spondin 3 expression and YAP levels remain elevated long-term, suggesting that the bacteria are able to interfere with the regulatory crosstalk between gland epithelial and stromal cells, thus causing a chronic regenerative state in the epithelial cells. This chronic, pro-regenerative response, which is seen in both antrum and corpus, could contribute to the establishment of permissive conditions that can ultimately result in neoplastic changes.

Wizenty and colleagues recently reported that in antral glands R-spondin 3 also acts *via* the Lgr4 receptor, activating not only Wnt, but also NF-κB signaling in stem and progenitor cells (83) upon direct interaction with *H. pylori* (**Figure 1**). Interestingly, another recent publication illustrated that NF-κB signaling and β-catenin activation (an important transcription factor for activating Wnt signaling) are connected *via* CDK1 upregulation upon *H. pylori* infection (84). *H. pylori* infected Lgr4 null mice did not develop gland hyperplasia and expressed lower levels of various chemokines such as CXCL1 and CXCL2, which are induced *via* NF-κB (83). *In vitro* experiments with gastric organoids further demonstrated that organoids enriched in progenitor-like cells (grown in the presence of R-spondin) were able to activate NF-κB signaling in response to ADP-heptose, while this was not the case in organoids differentiated into pit-like cells through removal of R-spondin from the medium. Similarly, previous studies have also reported that pro-inflammatory responses to *H. pylori* in organoids and mucosoids are enriched in gland base cells, rather than pit cells (85, 86). This implies that stem cells have a unique potential to elicit a strong pro-inflammatory response to bacteria-derived factors, suggesting that gland-colonizing bacteria may harbor a distinctive pathogenic capacity while bacteria located at the pit region are more tolerated by the host.

Taken together, these studies suggest that *H. pylori*, directly and indirectly, dysregulates important signaling pathways involved in gastric stem cell niche maintenance, leading to a persistent and uncontrolled regenerative state that can cause severe hyperplasia and sets the basis for malignant progression (**Figure 1**). Moreover, identifying the mechanisms that initiate the inflammatory response in a cell-type-dependent manner will be fundamental to broadening our knowledge of how the bacterial populations localized to different sites affect the host, causing chronic inflammation and tumorigenesis.

## 5 Summary and future prospective

Since its discovery, a myriad of studies has highlighted *H. pylori*’s cellular effects, ranging from DNA damage to altered cellular signaling. With the identification of a sub-population that can colonize deep in the glands has come the realization that *H. pylori* may directly damage stem cells and alter their genetic integrity. Furthermore, gland colonization causes a pro-regenerative reprogramming of the stem cell compartment, resulting in increased epithelial proliferation and stem cell self-renewal. Damage to long-lived cells that continually renew the glands and induction of a chronic regenerative stem cell state likely synergize to provide conditions that are permissive for the development of gastric cancer. We are now beginning to decipher the cell type-specific mechanisms underlying these processes.

Further investigations will be required to identify the exact synergistic pro-carcinogenic effects of mutational events induced by infection of stem cells and the *H. pylori*-driven changes in the stem cell microenvironment, focusing on how the microenvironment enables the formation, selection and expansion of cancer-initiating cell clones. Furthermore, there is also evidence that crypt or gland colonization occurs at other sites of the gastrointestinal tract. The concepts presented here, such as the biogeography of infection, the ability to invade and colonize crypts and the crypt cell-specific epithelial responses are likely relevant not only in the context of *H. pylori* infection but may represent fundamental determinants of pathogenicity of gastrointestinal host-microbe interactions (87).

## Author contributions

GB: literature research, drafting of the manuscript, creation of figure. MS: drafting and revision of manuscript and figure, supervision. All authors contributed to the article and approved the submitted version.
